# Increased amount of phosphorylated proinflammatory osteopontin in rheumatoid arthritis synovia is associated to decreased tartrate-resistant acid phosphatase 5B/5A ratio

**DOI:** 10.1371/journal.pone.0182904

**Published:** 2017-08-08

**Authors:** Jani Luukkonen, Laia Mira Pascual, Christina Patlaka, Pernilla Lång, Sanna Turunen, Jussi Halleen, Tomi Nousiainen, Maarit Valkealahti, Juha Tuukkanen, Göran Andersson, Petri Lehenkari

**Affiliations:** 1 Department of Anatomy, Medical Research Center Oulu, University of Oulu, Oulu, Finland; 2 Department of Laboratory Medicine, Division of Pathology, Karolinska Institutet, Stockholm, Sweden; 3 Pharmatest Services Ltd., Itäinen Pitkäkatu 4, FI, Turku, Finland; 4 Department of Surgery, University of Oulu Hospital, University of Oulu, Oulu, Finland; Universita Cattolica del Sacro Cuore, ITALY

## Abstract

**Background:**

Osteopontin (OPN) is an immunoregulatory protein which production increases in both rheumatoid arthritis (RA) and osteoarthritis (OA). Phosphorylated osteopontin (Phospho-OPN) is known to increase macrophage and osteoclast activation, this process is controlled by extracellular tartrate-resistant acid phosphatase (TRAcP), also a biomarker for RA. Here, we evaluated the phosphorylation status of OPN in RA and OA synovia, as well as its correlation with TRAcP isoforms.

**Methods:**

Synovial tissue and fluid were obtained from 24 RA (14 seropositive and 10 seronegative) and 24 OA patients. Western blotting was used to analyze the extent of OPN phosphorylation. TRAcP isoforms were measured in synovial fluid using ELISA; immunohistochemistry assessed the distribution of OPN and TRAcP expressing cells in the synovial tissue, especially distinguishing between the TRAcP isoforms.

**Results:**

Full-length OPN was more phosphorylated in RA than in OA (p<0.05). The thrombin cleaved C-terminal end of OPN was also more phosphorylated in RA (p<0.05). RA patients had a lower concentration of TRAcP 5B and higher concentration of less active 5A in their synovial fluid compared to OA patients. The TRAcP 5B/5A ratio was decreased in RA and correlated negatively with the amount of phospho-OPN (p<0.05). TRAcP positive cells for both isoforms were found all along the synovial lining; OPN antibody staining was localized in the extracellular matrix.

**Conclusion:**

Our data suggests that in RA the synovial fluid contains insufficient amounts of TRAcP 5B which increase levels of the proinflammatory phospho-OPN. This may lead to increased macrophage and osteoclast activation, resulting in the increased local inflammation and bone resorption present in RA joints.

## Introduction

Rheumatoid arthritis (RA) is a chronic autoimmune inflammatory disease characterized by pathological changes in joints such as synovitis, cartilage degradation, subchondral bone erosions, and increased secretion of proinflammatory cytokines [1]. RA patients can be divided into two subgroups, seropositive and seronegative, according to the level of rheumatoid factor or anti-citrullinated protein antibodies, with the seropositive group being positive for one of these biomarkers [2]. The seropositive group is a more homogenous group with a more similar disease etiology and more aggressive symptoms [3]. Generally it is thought that synovitis and inflammation in RA are primary effects whereas cartilage wear and periarticular osteolysis represent secondary phenomena.

Osteoarthritis (OA) is considered more as a disease of cartilage wear, believed to be caused by mechanical damage to synovial joint surfaces. Mechanical wear induces the production of proinflammatory cytokines in bone and cartilage, which leads to the development of a secondary chronic inflammation in the synovial tissue [4]. OA and RA share many pathological similarities but generally, the inflammation is weaker and possibly secondary in OA.

Osteopontin (OPN) is a glycoprotein capable of being phosphorylated, which was first identified in bone matrix, but later it has been discovered in almost all human tissues; today it is considered to be a soluble, although sometimes bound, extracellular matrix protein that has a signaling function through integrins and CD44 variants [5–7]. It is expressed in a large variety of cells such as bone cells i.e. osteoblasts, osteoclasts and osteocytes and also inflammatory dendritic cells and macrophages, as well as fibroblasts, chondrocytes, endothelial cells, smooth muscle cells etc. [5–7]. It has a total of 36 phosphoresidues, 34 phospho-serine and 2 phospho-threonine groups. *In vivo* OPN is phosphorylated intracellulary for example by Fam 20C kinase.[8] OPN is present in abundance in various tissues and it mediates cell migration, adhesion, activation and also many other cell functions. Importantly, its expression is elevated in inflammation and cancer, as it acts also as an important proinflammatory mediator, especially in macrophages and other phagocytic cells [7, 9]. This proinflammatory action is more pronounced with the phosphorylated form of the molecule. Phosphorylation of OPN is either essential or it enhances many of its primary functions, such as inflammatory cell and osteoclast activation, migration and differentiation, as well as inhibition of biomineralization [7, 10–12].

OPN is a major substrate for tartrate-resistant acid phosphatase (TRAcP); this enzyme can be considered as a key regulator of OPN’s major functions since it regulates the extent of phosphorylation of extracellular OPN [13, 14]. Two isoforms of TRAcP are found in the human body, TRACP 5A and 5B. The 5A form is a relatively inactive pro-enzyme, but proteolytic processing of the 5A enzyme, by members of the cathepsin family such as cathepsin K or other proteinases, into two-subunit of the 5B form increases its activity by at least ten fold [15–17]. In general, it is thought that the 5A form is secreted by macrophages and the 5B form by osteoclasts [18]. In both RA and OA, overall TRAcP levels in serum are elevated and furthermore TRAcP has been claimed to be a good marker of osteoclast number and macrophage activation [19–22].

OPN can be cleaved by thrombin into two active parts, its N- and C-terminal ends. Both of these molecules continue to function as proinflammatory cytokines, with their abilities being dependent on the extent of their phosphorylation. The C-terminal fragment induces macrophage chemotaxis and the N-terminal fragment cell spreading and activation [11]. Recent studies have detected elevations in the levels of OPN in RA and OA in both plasma and synovial fluid, and that the OPN levels correlate with TRAcP levels in RA [23, 24]. In OA, OPN levels have been shown to correlate with disease severity and an increase in the amount of phospho-OPN in cartilage has been claimed to increase its degradation [25, 26].

The goal of this study was to understand the relationship between OPN, and 5A and 5B TRAcP isoforms in OA and RA joint tissues. Therefore, we measured the levels of phosphorylated OPN and TRAcP isoforms in synovial fluid samples. Our hypothesis was that TRAcP 5A/B levels would differ significantly in OA and RA which may result in changes in the phosphorylation of OPN. Excessive phospho-OPN could then in part contribute to the chronic inflammation in RA, as it is known that phospho-OPN increases immune cell activation and inflammation. Another goal was to utilize immunohistochemistry to determine whether TRAcP is produced also in the synovial lining.

## Materials and methods

### Synovial fluid and tissue acquisition

Synovial fluid and biopsies were taken from 24 RA (14 seropositive and 10 seronegative) and 24 OA patients undergoing total knee replacement operation and synovectomy. All patients were from Finland Northern Ostrobothnia area. Patient data is shown in Table 1. Biopsies were taken from the anterolateral side of the joint capsule and cast in paraffin blocks for later histological analysis. RA patients were divided into seropositive and seronegative subgroups to determine if there was any difference between the different types of rheumatoid arthritis. All seropositive were positive for rheumatoid factor, and all but two were also positive for anti-citrullinated protein antibodies. As the seropositive group is believed to be a more homogenous group, it was predicted that there would be less intra-group variation in the protein measurements in this group. Clinical synovitis and excessive synovial fluid was evident in all patients as could be seen from the volume of acquired synovial fluid. The total mean volumes of acquired synovial fluid were 3.14 ± 0.35 ml from seropositive RA (RF+), 2.90 ± 0.35 ml from seronegative RA (RF-) and 4.05 ± 0.55 ml from OA patients. In our experience the volume of synovial fluid that can be acquired during surgery from healthy uninflamed knees is at maximum 1ml. No additional trauma was caused during sample acquisition. The patients were informed and they provided written approval for use of their samples. The protocol followed the Helsinki Declaration principles in full, and Northern Ostrobothnia Hospital District Ethical Committee provided an approval for the study and tissue collection (Statement 29/2011).

**Table 1:** Patient data.

	Gender	Average age (years)	Time since diagnosis (years)	Disease-modifying antirheumatic drugs	Biological drugs
**RF+**	12 female 2 male	69.21 ± 7.93	25.00 ± 10.88	13	2
**RF-**	8 female 2 male	70.90 ± 6.33	27.11 ± 12.75	6	
**OA**	18 female 6 male	70.31 ± 8.30			

### Phospho-OPN measurement

Western blot analysis of the synovial fluid samples was done to determine the relative level of total and phospho-OPN in RA and OA patients’ synovial fluid. Four 12% 15-well SDS-polyacrylamide gels were made, and on each, two gels were loaded with equal amounts of 20 synovia samples. One OA synovia sample had to be omitted due to a poor quality sample. After electrophoresis, the sample proteins were transferred onto Protran nitrocellulose membranes, and it was checked that there were equal gross protein loads with Ponceu S dye staining. The membranes were blocked using blocking buffer containing 4% milk and 0.05% TWEEN in PBS. First two gels were incubated with osteopontin antibody, which binds to full-length and the C-terminal fragment of OPN, (1:1000, Acris Antibodies, San Diego, CA, USA) followed by horseradish peroxidase conjugated anti-rabbit IgG secondary antibody (1:2000, Bio-Rad). Later two gels were treated with a phosphoserine antibody, which binds to all phosphorylated serine amino-acids, (1:500, Sigma-Aldrich) followed by horseradish peroxidase conjugated anti-mouse IgG secondary antibody (1:2000, Bio-Rad). All four membranes were treated with an enhanced chemiluminescent substrate for the detection of horseradish peroxidase on the immunoblots (Pierce ECL Western Blotting Substrate, Thermo Scientific) and exposed to a high performance chemiluminescence film (Amersham Hyperfilm ECL, GE Healthcare). Since there is no antibody on the market which detects only the phosphorylated form of OPN, OPN and phosphoserine films were compared by setting them on top of each other on a light table to determine the location of phospho-OPN bands on the phosphoserine films. To quantify the relative concentrations of total and phospho-OPN, the relative optical densities (RODs) of the bands were measured using MCID Core Digital Imaging Software (MCID, United Kingdom). The total OPN was obtained by measuring all full-length OPN band densities.

#### TRAcP 5A/B measurement

96-well ½ volume ELISA plates (Corning, Corning, NY) were coated with 5 μg/ml (50μl/well) mAb 46 (specific for TRAcP 5A; Mabtech, Nacka, Sweden) or mAb25.44 (recognising TRAcP 5A and 5B; Mabtech) diluted in PBS at 4°C overnight. Samples and standards were pre-treated as follows: samples were diluted 1:1 and incubated with 1.5 M glycine pH 2.3 for 1h at 37°C. After incubation, the samples were neutralized by addition of 1 volume 1M Tris-HCl pH 8.5 leading to final dilution of serum samples of 1:3. mAb46 plates were washed 3 times in TBST (25mM Tris pH 7.4, 150mM NaCl, 0.1% Tween-20). Standards and samples (50μl/well), diluted 1:2 in ELISA buffer (Mabtech), were added to mAb46 and incubated overnight at room temperature. The samples were then transferred to the mAb 25.44 plate for TRAcP 5B detection. In the TRAcP 5B detection, mAb25.44 plates were incubated with transferred standards, new standards and samples for 2h at room temperature. For detection of TRAcP 5A (mAb 46 plates) and TRAcP 5B (mAb25.44) were washed 3 times in TBST and incubated with 0.25 μg/ml biotinylated detection mAb 12.56 (recognising TRAcP 5A and 5B; Mabtech) (50μl/well) diluted in 0.1% BSA+TBST for 1h at room temperature. Plates were washed 3 times in TBST and incubated with streptavidin-HRP (Mabtech) diluted 1:1000 in 0.1% BSA+TBST for 1h at room temperature. Plates were washed 3 times in TBST and developed using K-Blue® Substrate (TMB) (Neogen, Lansing, MI) and the reaction was stopped by addition of 1M H_2_SO_4._ Absorbance was read at 450nm using BioTek’s PowerWave HT microplate spectrophotometer (BioTek, Winooski, VT). Recombinant TRAcP 5A standards were used to calculate the concentration of TRAcP 5A in the samples. In the assessment of the TRAcP 5A amount, any transferred TRAcP 5a was excluded by background extraction based on transfer of recombinant TRAcP 5A standards from the mAb46 to the mAb25.55 plate.

### Data analysis

Statistical analysis and plot figures were made with IBM SPSS Statistics 20 (IBM, NY, USA). P-values were assessed using non-parametric tests (Mann-Whitney for continuous variables), as the number of samples was limited so the distribution of the measures could not reliably be considered to be normal.

### Immunohistology

Immunohistochemistry was conducted to demonstrate TRAcP and OPN expression in synovial tissue. Paraffin was removed and the tissue samples were rehydrated through a xylene-ethanol series. TRAcP positive cells were stained with Dako EnVision+ System-HRP kit according to the manufacturer’s instructions using primary antibodies recognizing both TRAcP isoforms 5A and 5B (generic TRAcP) and a specific antibody for 5A isoform in 1:100 dilution. TRAcP antibodies were produced as described previously [15, 27] OPN antibody (Acris Antibodies, San Diego, CA, USA) was used in 1:200 dilution. Counterstaining was done with Mayer’s hematoxylin. To eliminate the possibility of confounding by non-specific binding of secondary antibody to tissues, the protocol was done also without primary antibodies as control; no staining was seen in the negative control samples. The relative staining intensities were quantified visually using a light microscope.

## Results

### Levels of phospho-OPN and TRAcP in synovial fluid

**Fig 1:**
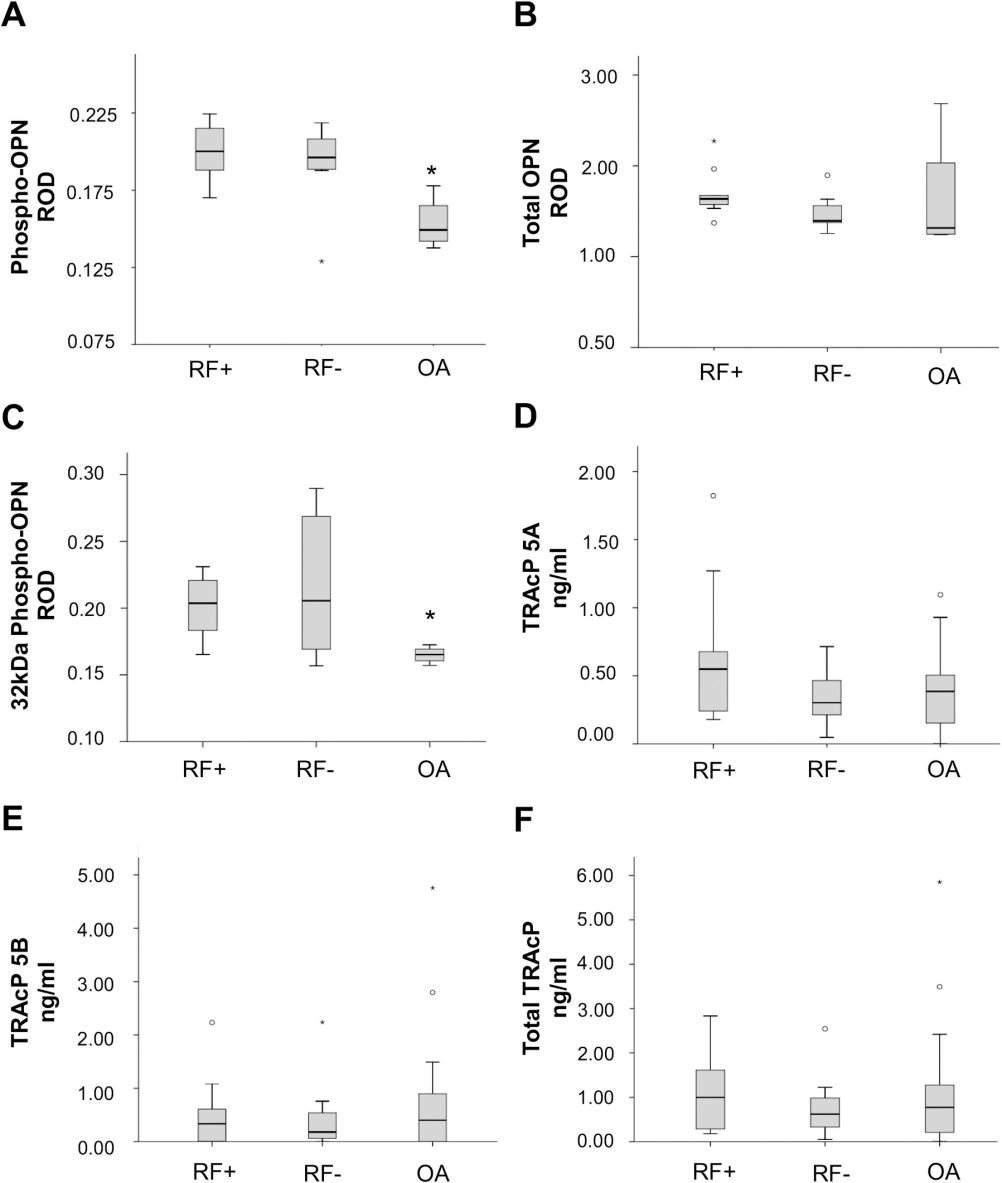
Schematic illustrations of phospho-OPN and TRAcP isoforms in synovial fluid. Relative optical densities (RODs) of western blot bands for phospho-OPN (A), summarized total OPN (B) and thrombin cleaved 32kDa phospho-OPN (C), with the measured TRAcP 5A and 5B levels (D and E) and the estimated total TRAcP (F) in synovial fluid from seropositive (RF+) and seronegative (RF-) RA and OA patients. Statistically significant differences are marked with an asterisk.

The immunoblotting method was used to determine the level of phosphorylated OPN present in the synovial fluids. The relative optical densities (ROD) of the bands were measures with an image analysis system. Illustrations of OPN and TRAcP levels are shown in Fig 1.

The level of full-length phospho-OPN in synovial fluid of seropositive (mean ROD 0.200±0.018, median 0.198) and seronegative (mean ROD 0.192±0.027, median 0.194) RA patients was higher when compared to the levels of OA patients (mean ROD 0.153±0.017, median 0.149), no significant difference between seropositive and seronegative group was seen, as shown in Fig 1A. The differences between all RA and OA patients were considered statistically significant, p<0.05. No differences were found between the levels of total full-length OPN between the patient groups, as shown in Fig 1B.

In addition, the level of phosphorylated C-terminal end of OPN fragment was higher in both the seropositive (mean ROD 0.201±0.024, median 0.204) and the seronegative (mean ROD 0.217±0.053, median 0.206) RA patients than in the OA patients (mean ROD 0.165±0.006, median 0.165), p<0.05, and there was no significant difference between seropositive and seronegative groups, as shown in Fig 1C.

TRAcP 5A (Fig 1D) and 5B (Fig 1E) concentrations were measured separately from synovial fluid. Twelve samples (3 RF+, 2 RF- and 7 OA) fell under the detection level. For statistical analyzes they were given a value of 0.001, which is well under the limit of detection. The mean level of TRAcP 5A in synovial fluid for seropositive RA was 0.640±0.130 (median 0.550) ng/ml, for seronegative RA 0.334±0.059 (median 0.303) ng/ml and for OA 0.393±0.056 (median 0.385) ng/ml. The mean levels for TRAcP 5B were 0.488±0.161 (median 0.334) ng/ml for seropositive RA, 0.463±0.213 (median 0.178) ng/ml for seronegative RA and 0.717±0.221 (median 0.400) ng/ml for OA.

A trend could be seen in the differences of TRAcP 5A and 5B between the disease groups. Seropositive RA patients had more TRAcP 5A than seronegative RA or OA patients, as OA patients had more TRAcP 5B than either of the RA patient groups. There were no significant differences between the estimated total TRAcP levels (Fig 1F).

**Fig 2:**
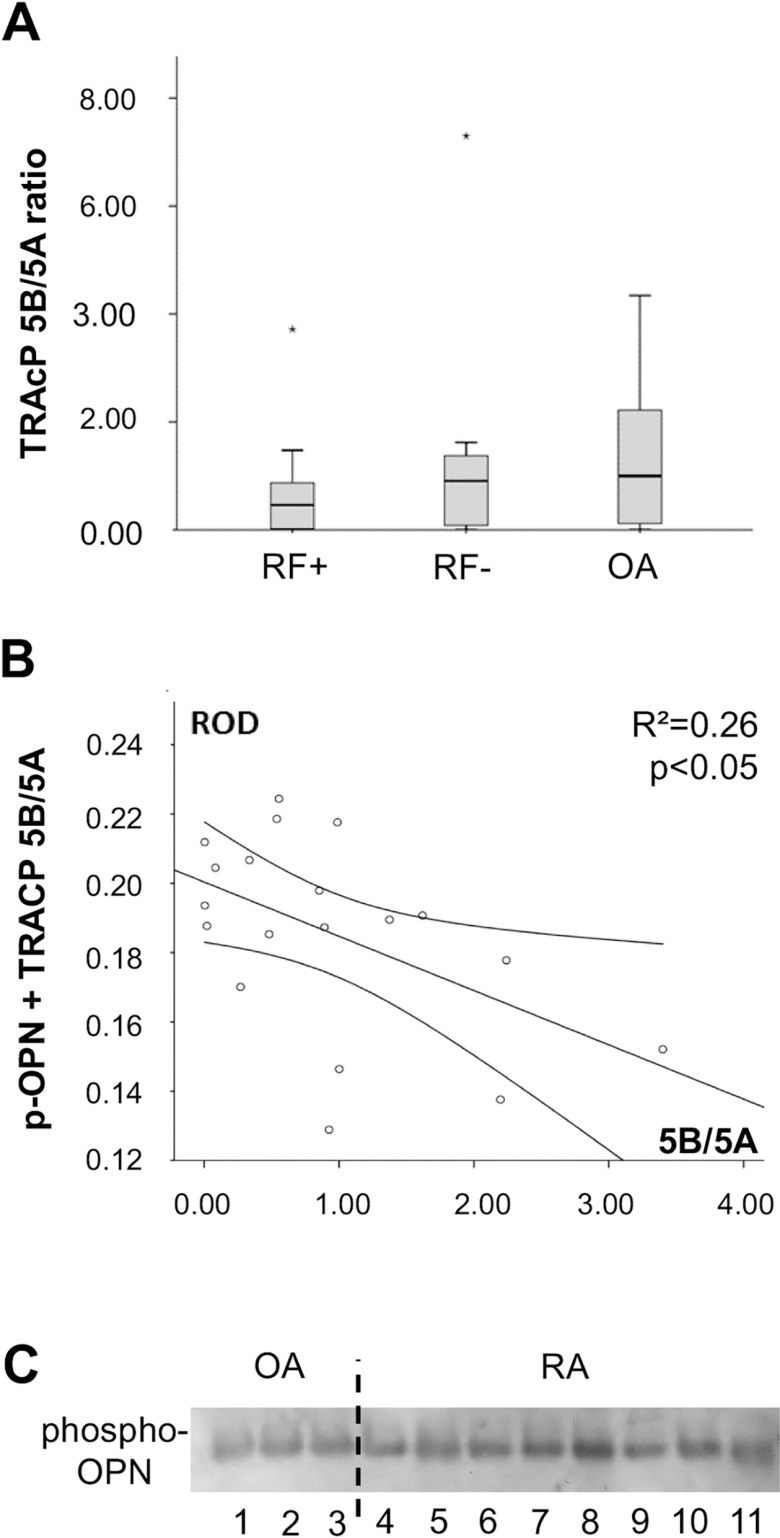
Summary of phospho-OPN and TRAcP isoform data. The TRAcP 5B/5A ratio (A) is lowered in RF+ RA and this correlates with the level of OPN’s phosphorylation (B), since TRAcP 5B is the primary phosphatase of OPN, its phosphorylation is decreased in RA. An image of phospho-OPN staining on western blot (C) is shown to highlight the differences, the photo was cropped to allow better visualization of the bands. In conclusion, we demonstrate that OPN is significantly more phosphorylated in RA than in OA synovia, and there are no significant differences between seropositive and seronegative groups (Fig 1A). TRAcP 5B/5A ratio in synovial fluid is increased in OA, and there is a significant negative correlation between the measured ratios and phospho-OPN levels.

When comparing the ratios of the two TRAcP isoforms (5B/5A) a pronounced difference (p = 0.07) could be noted between seropositive RA (mean ratio 0.714±0.259, median 0.459) and OA (mean ratio 1.384±0.275, median 1.000) patients; the ratios of the seronegative RA patients (mean ratio 1.388±0.680, median 0.909) were on the same level as OA patients but their distribution was more widespread than in OA (RF- standard deviation 2.152, OA 1.35) (Fig 2A).

Spearman correlation was calculated between TRAcP 5B/5A ratio and full-length phospho-OPN measurements to reveal possible correlations between the proteins in the synovia samples (Fig 2B). A significant negative correlation between the protein isoforms was found (r = -0.458, p<0.05) indicating that the higher TRAcP 5B levels result in lower phosphorylation of OPN. In addition, a significant positive correlation was found between the two TRAcP isoforms (r = 0.683, p<0.05) in all samples, as expected as TRAcP 5B is proteolytically processed from 5A.

### Immunohistology

**Fig 3:**
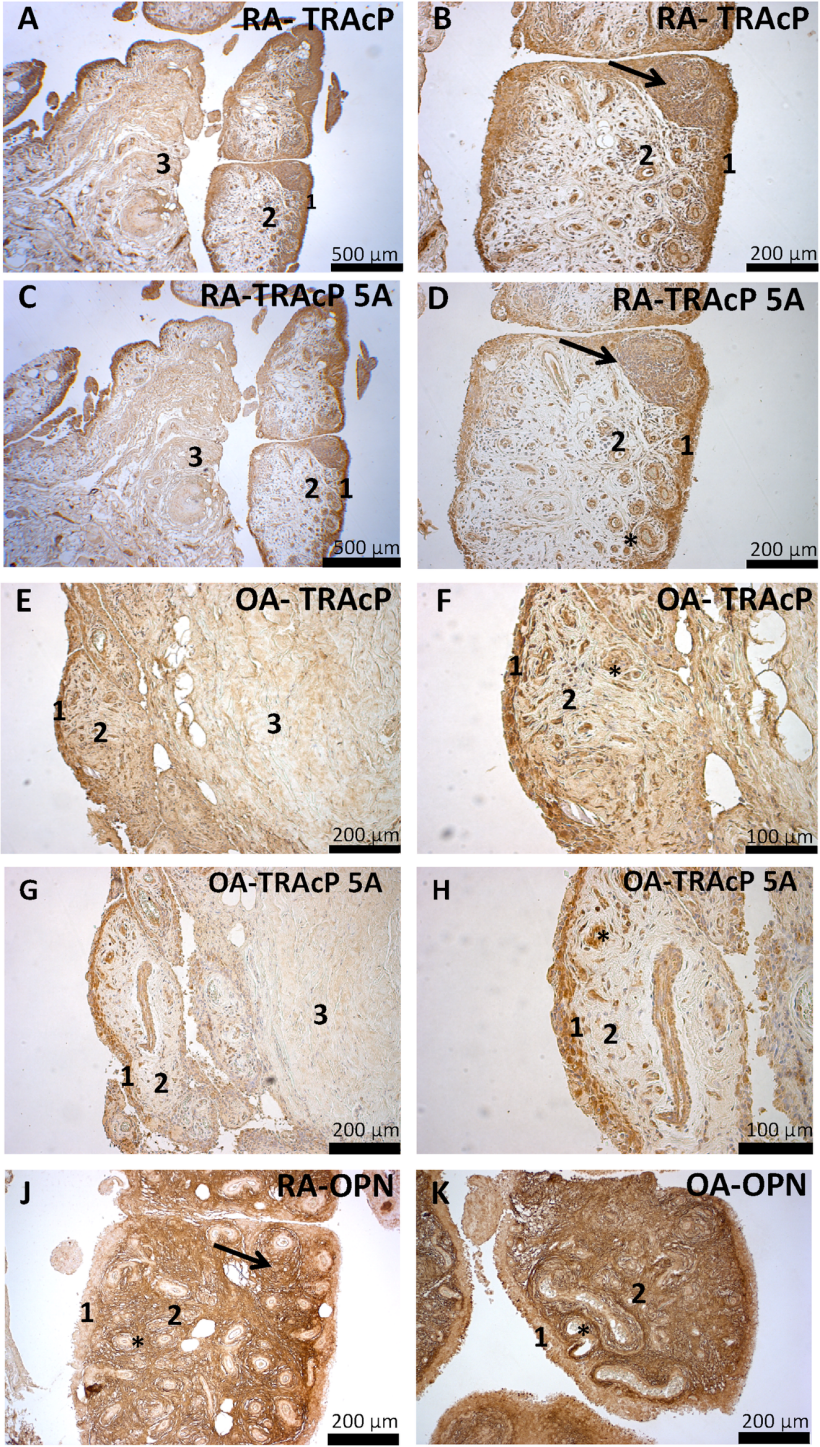
Synovial tissue immunohistology. The antibody staining in each panel is shown as brown color. The generic TRAcP antibody (A, B, E, F), which stains both 5A and 5B isoforms, localized mostly in the lining layer (1) cells in a similar pattern between RA and OA, but a slightly more intensive staining of the sublining layer (2) cells and stroma could be seen in OA samples. The pattern of staining within deep stroma (3), which consists mostly of dense connective tissue and fat, was also similar between the diseases. TRAcP 5A stain (C, D, G, H) localized similarly to the generic antibody, and no differences were found between staining intensities of the sample groups. This indicates that most of TRAcP in the synovial tissue is likely in the 5A form, but differences in TRAcP 5B levels within the synovial tissue are possible between OA and RA. Both TRAcP antibodies were also localized in endothelial (asterisk) and lymphatic cells within the lymphatic follicle (arrow). OPN antibody stain (J, K) localized in the extracellular matrix, with only slight intracellular staining seen. OPN staining was most intense in the sublining layer (2), while the stainings of the lining layer (1) and deep stroma were lighter. OPN antibody did not localize in the endothelial cells (asterisk) but was found within the lymph node (arrow) stroma and some cells within it. No difference was found in the pattern or intensity of staining between RA and OA samples.

Immunohistochemistry was used to show TRAcP expression in synovial tissue. Positive cells for both isoforms binding generic TRAcP antibody and for TRAcP 5A specific antibody could be found in a generally similar pattern, especially in the synovial lining. Endothelial and lymphatic cells also exhibited also strong staining with both antibodies. Lighter positive staining was also found in the sublining layer and deeper within the connective tissue. The similar staining pattern displayed by the two antibodies indicates that most of the TRAcP enzyme in the synovial tissue is likely in the 5A form. Relative staining intensities are shown in Table 2. Fig 3 shows example images of the synovial tissue histology. Quantification example is shown in the supplementary file S3 Fig.

**Table 2:** Relative intensities of staining.

	lining layer	sublining layer	deep stroma
**Generic TRAcP OA**	+++	++	++
**Generic TRAcP RA**	+++	+	++
**TRAcP 5A OA**	+++	+	++
**TRAcP 5A RA**	+++	+	++
**OPN OA**	+	+++	+
**OPN RA**	+	+++	+

When comparing RA and OA samples, a difference could be detected in the sublining layer staining with the generic TRAcP antibody. In OA samples, both cells and stroma in the sublining stained more strongly than in RA. With the 5A antibody, the pattern of staining was similar in both diseases. This indicates that there is likely more TRAcP 5B in OA synovial tissue in the sublining layer, a finding consistent with the higher 5B level in OA synovial fluid.

OPN staining was localized in the extracellular matrix. Only minor intracellular staining was found in the lining and sublining layer cells. The staining intensity was strongest in the sublining layer. Lighter staining was observed in the lining layer and deep stroma. No differences were detected between the diseases. No OPN staining was found in the endothelial cells. Lymphatic cells within stroma and lymph nodes, especially cells which showed macrophage like morphology, were stained positive.

## Discussion

The goal of this study was to assess in detail the relation of OPN’s phosphorylation and TRAcP 5A/B isoenzyme concentrations in RA and OA and to distinguish possible differences between these two clinically very different diseases. The main finding emerging from this study is that OPN is more phosphorylated in synovial fluid from RA patients in compared with the corresponding samples from OA patients. This correlates well with decreased TRAcP 5B/5A ratio. Previous studies concerning OPN have mostly focused on OPN production in different diseases and disease severities, i.e. increased OPN levels have been shown to correlate with a more severe disease phenotype [24, 25, 28]. OPN’s phosphorylation is known to exert a critical effect on a wide range of its cytokine properties from inhibition of biomineralization to osteoclast activation and pathogenesis of inflammatory diseases; this is because phosphorylation is required for its key functions, such as working as a proinflammatory cytokine [10–12, 26, 29–31]. The clinical significance of OPN’s phosphorylation has not been assessed in this context before.

**Fig 4:**
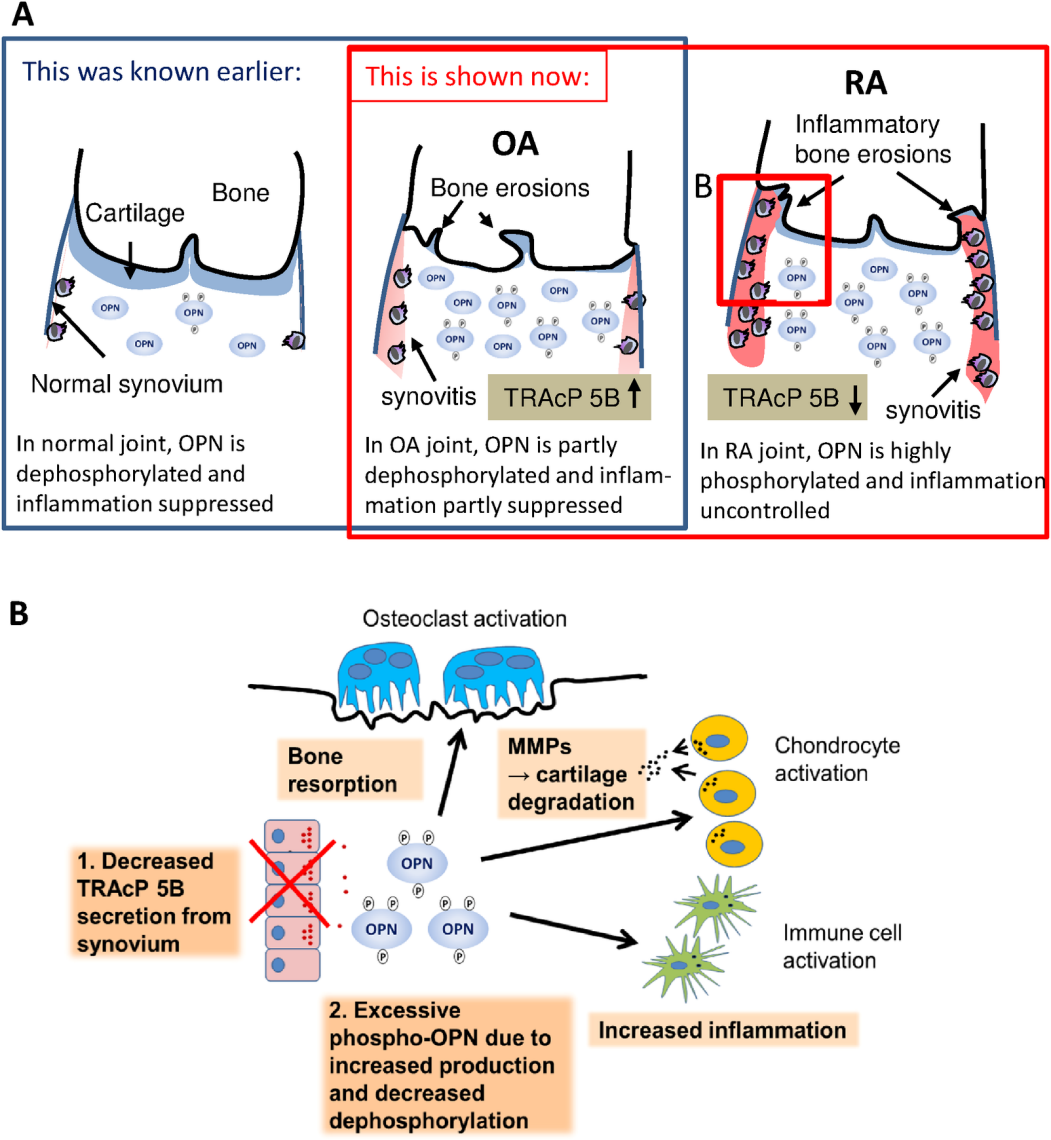
Schematic presentations of OPN’s role in OA and RA. Picture A displays a schematic illustration of the normal, osteoarthritis and rheumatoid arthritis synovial cavity. The phosphorylation of OPN in synovial fluid gradually increases from healthy to RA in correlation with the severity of the inflammation and symptoms. Picture B is an illustration of possible cell level actions influenced by OPN in the pathogenesis of RA. Insufficient TRAcP 5B production in synovial tissue may lead to an excessive phospho-OPN concentration, which leads to elevated immune cell activation, more cartilage destruction and greater activation of bone resorbing osteoclasts, all effects contributing to the pathogenesis of RA.

However, previously OPN has been shown to be more phosphorylated in synovia from OA patients when they have been compared to healthy controls, and by combining that data to our current results, we believe that there is an even greater difference in phosphorylation of OPN between the healthy and RA patients. [26] The extent of phosphorylation of osteopontin is likely modulated for different purposes in different tissues, for example, it has been shown in murine cells that the phosphorylation level OPN may differ depending on the secreting cell type. [32] Our data suggests that more extensive phosphorylation of OPN is linked to a low TRAcP 5B concentration in synovial fluid, but another possible hypothetical mechanism functioning at the same time is that the synovial cells of RA patients produce a more phosphorylated form of OPN than cells of individuals with OA. It is noteworthy that our synovial samples originate from patients undergoing total knee prosthesis operation due to joint destruction. Hence, in both disease groups, their inflammatory stimulus is evident. The proinflammatory cytokine profile of these OA synovial fluid samples is likely closer to RA than in early stages of OA, but the heterogeneity of samples is reduced as the patients have to have met strict criteria for knee prosthesis operation. As phospho-OPN, both full-length and the C-terminal end, induce macrophage activation and thereby inflammation more than the dephosphorylated form of OPN, the high concentrations of phospho-OPN in RA synovial fluid and tissue under these circumstances may represent one possible pathogenic mechanism behind the chronic inflammatory disease or as is often observed in RA patients, the related bone destruction. [11] Fig 4 is a schematic representation of our findings and hypothesis (A) as well as its possible contribution to the pathological mechanisms involved in RA (B).

TRAcP enzyme is a marker of bone resorption, and its production is known to be up-regulated in both OA and RA [20, 22]. In this study, our data suggests that the levels of the TRAcP 5A and 5B isoforms are different in RA and OA. We hypothesize that this is a reflection of the different pathogenetic processes underpinning RA and OA by regulating the phosphorylation of OPN. Our data suggests further that the TRAcP 5B/5A ratio correlates with the level of OPN’s phosphorylation in OA and RA. There is more of the 5B isoform in OA, which in turn could lead to lower phospho-OPN levels via more OPN dephosphorylation. One explanation to account for this phenomenon is that the production of phospho-OPN is upregulated during inflammation, and if there is an insufficient TRAcP 5B level in synovia, this would lead to an excess phospho-OPN concentration, which would evoke a stronger inflammatory process, and hence it could play a part in RA pathogenesis. As phospho-OPN is known to increase macrophage and osteoclast activation, this may result in cartilage and bone destruction, and enhanced synovitis. Together with the known increases in other pro-inflammatory cytokines, the pathological activation of inflammatory cells also leads to an increased production of OPN [33]. OPN is a factor in creating a vicious circle with chronic inflammation representing the driving force.

In previous studies, TRAcP producing mono and multinucleated cells have been identified in OA and RA synovial tissue. [19, 34] Our results support these findings that at least mononucleated TRAcP positive cells can be found in synovial tissue, as can be seen in Fig 3. This suggests that TRAcP is produced in the synovial tissue. Interestingly, we have also detected multinucleated cells in synovial tissue cell cultures for other studies. We observed that both isomers can be detected in the synovial tissue, and most of the TRAcP is likely present in its 5A form. TRAcP 5A is proteolytically processed into 5B form by cathepsin K and other proteinases, and it has been shown that the levels of these proteinases are higher in RA than in OA synovium and synovial fluid. [35, 36] In relation to our data, it seems that there could be some mechanism which prevents TRAcP 5A from being processed in RA or the conversion mechanism does not function properly. It would be interesting in future studies to determine what type of synovial cells produce TRAcP. One could speculate that most of the TRAcP positive cells are macrophage-like synoviocytes, but the diffuse staining of the cells suggests that fibroblast-like synoviocytes may also be TRAcP positive. This raises a question on the role of TRAcP for fibroblast-like synoviocytes and its origin.

During this study, we discovered that the synovial tissue did not stain when using TRAcP enzyme detection kit, which utilizes enzyme activity. One technical explanation is that the enzyme structure may have been damaged during paraffinization, as we have used the same kit for other histological studies without paraffin embedding, but some non-artefactual mechanism cannot be excluded, especially as it seems that most of TRAcP in synovial tissue is in the less active 5A form, which does not stain i.e. the TRAcP 5A does not give signal for enzyme activity staining using the enzyme method. In future studies on TRAcP histology, we suggest that instead of enzyme histochemistry, immunohistochemistry should be applied, as was done in this study.

The low number of study subjects and a lack of healthy control group are the most important limiting factors for this study. At the moment, we do not have ethical approval for acquiring synovial samples from healthy individuals and this limits any acquisition of material from healthy patients. With a larger study population, statistical significances would likely be more evident, as clear differences can be seen in the figures, nevertheless even with the current sample number, the results did reach statistical significance. A technical limitation is also the lack of specific phospho-OPN antibody, as the use of a different antibody to distinguish the phospho-OPN band on the blot film may decrease the method’s specificity. In addition, it would be beneficial if the detection level for TRAcP isoenzymes could be lower to avoid exclusions; we hope to improve the technique’s sensitivity in future studies. We also hope to include more patients in a possible follow-up study. If this can be achieved, then the link between TRAcP positive cells in synovial tissue and pathological bone resorption in both OA and RA may become clearer.

## Conclusion

As far as we are aware, this is the first study to measure differences in the level of phosphorylation of osteopontin in RA and OA patients’ synovial fluid. We observed that the level of phosphorylated OPN was significantly higher in RA than OA patients’ synovial fluid and the concentrations of TRAcP isoforms, less active 5A and active 5B, were in accordance with the phosphorylation of OPN. We also demonstrated that TRAcP is produced in the lining layer of synovial tissue, from where it can be secreted into synovial fluid. In summary, this data combined with earlier literature indicates that OPN and modulation of its phosphorylation could be an important component of the inflammatory reaction in RA.

## Supporting information

S1 Fig Osteopontin western blot stain. (TIF)

S2 Fig Phosphoserine western blot stain. (TIF)

S3 Fig Immunohistology quantification example. (TIF)
