# Supplementary figures and images for: Increased amount of phosphorylated proinflammatory osteopontin in rheumatoid arthritis synovia is associated to decreased tartrate-resistant acid phosphatase 5B/5A ratio
Source: PLoS One. 2017 Aug 8;12(8):e0182904. doi: 10.1371/journal.pone.0182904 (PMC5549736; doi:10.1371/journal.pone.0182904)

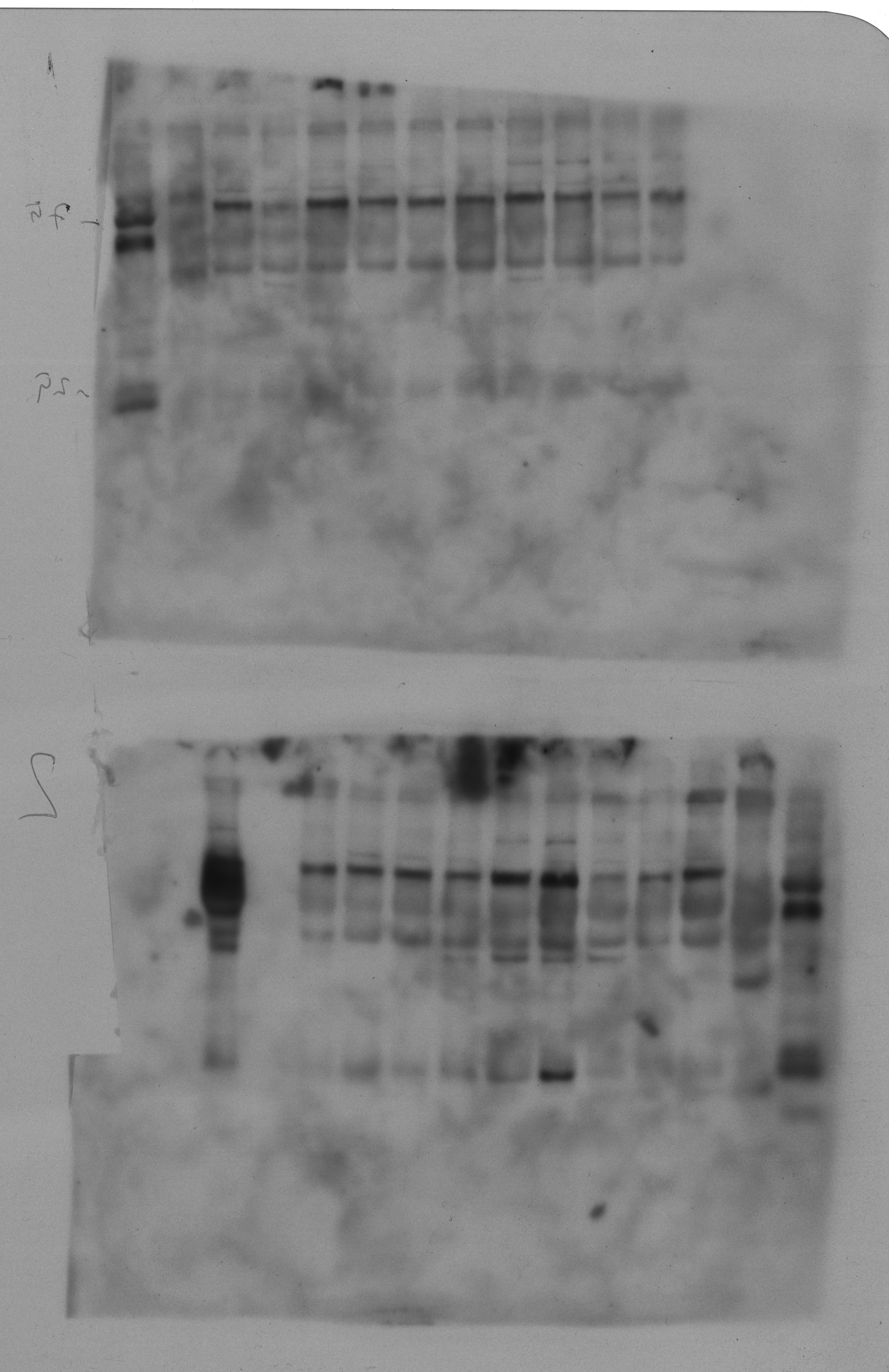

Supplement: S1 Fig — (TIF) [file pone.0182904.s001.tif]

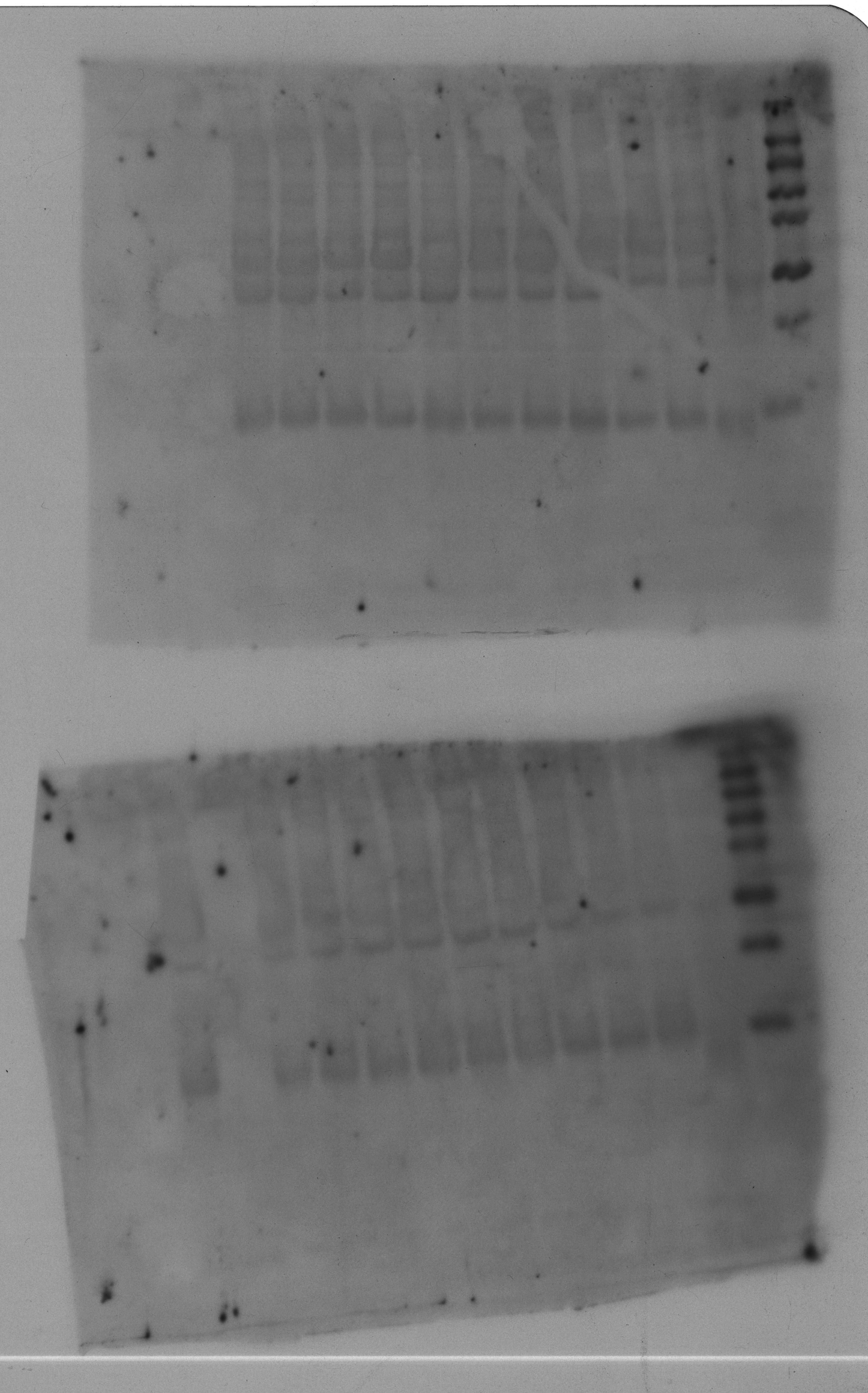

Supplement: S2 Fig — (TIF) [file pone.0182904.s002.tif]

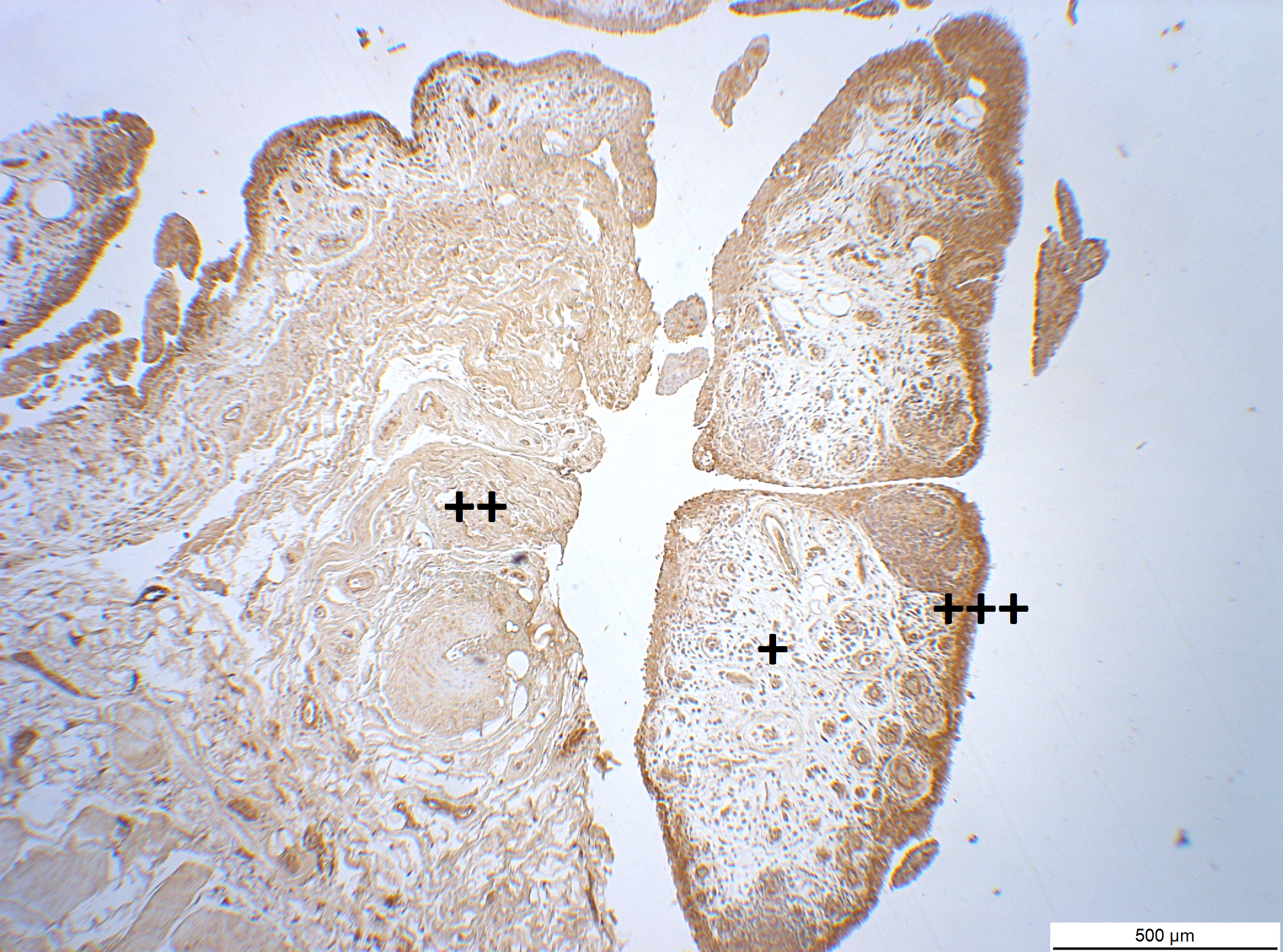

Supplement: S3 Fig — (TIF) [file pone.0182904.s003.tif]
